# Current progress and future perspectives in the development of anti-polo-like kinase 1 therapeutic agents

**DOI:** 10.12688/f1000research.11398.1

**Published:** 2017-06-29

**Authors:** Jung-Eun Park, David Hymel, Terrence R. Burke, Jr., Kyung S. Lee

**Affiliations:** 1 Laboratory of Metabolism, Center for Cancer Research, National Cancer Institute, National Institutes of Health, Bethesda, MD, 20892, USA; 2 Chemical Biology Laboratory, Center for Cancer Research, National Cancer Institute at Frederick, Frederick, MD, 21702, USA

**Keywords:** anti-cancer therapeutics, anti-mitotic agents, mitotic targets, polo-like kinase 1, Plk1, anti-Plk1 agents

## Abstract

Although significant levels of side effects are often associated with their use, microtubule-directed agents that primarily target fast-growing mitotic cells have been considered to be some of the most effective anti-cancer therapeutics. With the hope of developing new-generation anti-mitotic agents with reduced side effects and enhanced tumor specificity, researchers have targeted various proteins whose functions are critically required for mitotic progression. As one of the highly attractive mitotic targets, polo-like kinase 1 (Plk1) has been the subject of an extensive effort for anti-cancer drug discovery. To date, a variety of anti-Plk1 agents have been developed, and several of them are presently in clinical trials. Here, we will discuss the current status of generating anti-Plk1 agents as well as future strategies for designing and developing more efficacious anti-Plk1 therapeutics.

## Introduction

For several decades, anti-microtubule (MT) drugs such as taxanes and vinca alkaloids have been effectively used against a wide range of cancers, including solid and hematological malignancies ^1^. However, one of the major shortcomings of these MT-targeting agents has been severe and dose-limiting side effects that arise as the consequence of indiscriminately disrupting widespread MT functions, not only in actively dividing mitotic cells but also in non-dividing interphase cells. Thus, over the past decade, a high level of interest has been drawn to targeting a variety of mitosis-specific proteins in order to develop agents that can specifically disrupt the mitotic progression of highly proliferative cancer cells. These proteins include protein kinases (polo-like kinase 1 [Plk1] ^2^ and Aurora A ^3^), motor proteins (CENP-E ^4, 5^ and Eg5 ^6^), DNA-damage checkpoint proteins (Chk1 and Chk2 ^7^), and components of the ubiquitin proteasome pathway (APC/Cdc20 and the proteasome ^8, 9^). Among these endeavors, anti-Plk1 drug discovery has reached an advanced stage of development that merits reflection on its progress. In this short review, we will summarize recent advances and future directions toward developing therapeutics against one of the most appealing anti-cancer drug targets, Plk1.

## Plk1 as an anti-mitotic target

**Figure 1.**
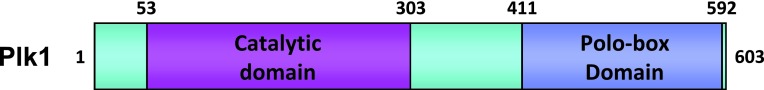
Schematic diagram of human polo-like kinase 1 (Plk1). The numbers indicate the positions of the amino acid residues in human Plk1.

Plk1 belongs to the polo subfamily of Ser/Thr protein kinases (collectively, Plks) and plays a key role at multiple stages of mitotic progression ^10^. Plk1 is composed of the *N*-terminal catalytic domain and the *C*-terminal non-catalytic polo-box domain (PBD) ( Figure 1). The cooperative action of these two domains is critical for Plk1 to regulate diverse mitotic processes ^11^. Not surprisingly, Plk1 is overexpressed in a wide spectrum of human cancers ^12^, and its overexpression is thought to promote genomic instability and tumorigenesis ^13– 15^. In addition, upregulated Plk1 activity appears to be closely associated with the aggressiveness and poor prognosis of these cancers ^16, 17^. Other studies have shown that various cancer cells—but not their isogenic normal cells—are addicted to Plk1 overexpression for their viability ^18– 20^. Since reversing addicted protein functions has proven to be an attractive strategy to selectively kill cancer cells ^18, 21– 23^, addiction to overexpressed Plk1 exacerbates the vulnerability of cancer cells to Plk1 interrogation. Thus, targeting Plk1 may permit the induction of cancer-cell-selective mitotic block and apoptotic cell death in Plk1-addicted cancers ^24^. Because human cancers are frequently slow growing, inhibiting a cancer-addicted target, such as Plk1, could be particularly effective in achieving the full therapeutic potential of an anti-mitotic agent.

## Promising Plk1 ATP-competitive inhibitors and their limitations

**Figure 2.**
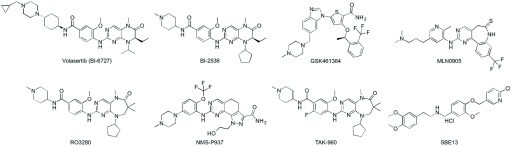
The structures of polo-like kinase 1 (Plk1) catalytic domain inhibitors. Only widely studied inhibitors are shown.

Targeting the catalytic activity of a protein kinase has been the predominant method of generating kinase inhibitors. Accordingly, a large number of ATP-competitive inhibitors directed against the catalytic activity of Plk1 have been developed and tested under various preclinical and clinical settings ^24^ ( Figure 2). Among them, volasertib (a dihydropteridinone derivative; Boehringer Ingelheim) is widely considered the most advanced inhibitor in this class, exhibiting potent anti-tumor activities in multiple nude mouse xenograft models ^25^. Volasertib has also shown significant clinical efficacies against advanced solid and hematological cancers in phase I/II clinical trials ^26– 30^. However, the initial outcome of its phase III clinical trials, performed with a cohort of elderly acute myeloid leukemia patients, turned out to be less than satisfactory (the 21st Annual Congress of the European Hematology Association, 2016). In addition, several other ATP-competitive inhibitors, such as GSK461364 (a thiophene derivative; GlaxoSmithKline) ^31^, MLN0905 (a benzolactam derivative; Millennium) ^32, 33^, RO3280 (a pyrimidodiazepine derivative; Roche) ^34, 35^, NMS-P937 (a pyrazoloquinazoline derivative; Nerviano) ^36, 37^, and TAK-960 (a 2-aryl pyrimidodiazepinone derivative; Takeda) ^38^ have shown only limited efficacy with more-than-acceptable dose-limiting toxicity in diverse preclinical/clinical trials. Dose-limiting toxicity arises mainly from non-specific activity of the inhibitors ^39^. In fact, one of the common problems associated with the currently available Plk1 ATP-competitive inhibitors is their low degree of selectivity against other kinases ^24^, including two that are closely related, Plk2 and Plk3, with possible tumor-suppressor function ^40, 41^. Therefore, improving Plk1 specificity is likely one of the most pressing concerns to address in order to accomplish better clinical outcomes with fewer toxicological problems.

## Future strategies to conquer current obstacles

Generating ATP-competitive inhibitors aimed at inhibiting the catalytic activity of a protein kinase is a widely used approach, in part, because isolating small molecules that block the catalytic activity of a kinase is relatively straightforward. However, owing to the highly conserved nature of the ATP-binding pocket, a large fraction of this class of inhibitors show a significant level of cross-reactivity with various other kinases ^39^. Therefore, for a given kinase, having a better understanding of the exact nature of the ATP-binding site (and its neighboring interaction pockets, if they exist) could be crucial for overcoming lack of specificity associated with non-allosteric ATP-competitive inhibitors.

### Improving specificity through structure-assisted optimization

**Figure 3.**
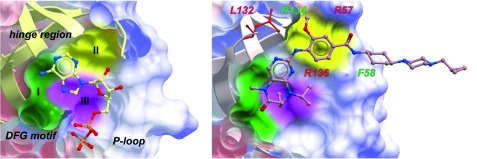
The binding modes of ADP (left panel, PDB: 3D5W) and volasertib (right panel, PDB: 3FC2) to the catalytic domain of polo-like kinase 1 (Plk1). Ligand-binding regions that are important for achieving kinase selectivity are shown as colored surfaces. Region I is located near the conserved Asp-Phe-Gly (DFG) motif, which is important in the design of certain allosteric inhibitors. Region II is near the hinge region, and region III is a relatively hydrophobic pocket below the adenine-binding site. Compared to Plk2 and Plk3, residues that are specific (green) or semi-specific (red) to Plk1 are labeled. Notably, the aryl-methoxy group in region II (near L132 of the hinge region) is used to produce moderate selectivity for Plk1 against other Plks ^44^.

Further development of Plk1 ATP-competitive inhibitors can be achieved through structure-guided medicinal chemistry targeting unique residues lining the ATP-binding pocket. Comparative analyses of the X-ray co-crystal structures of the catalytic domains of Plk1–3 revealed that the overall shapes of their ATP-binding pockets are similar, and the residues forming the pockets are mostly analogous to one another. Nevertheless, both F58 and R134 residues are unique to Plk1, whereas R57, L132, and R136 residues found in Plk1 and one of Plk2 and Plk3 ATP-binding pockets are semi-specific to Plk1 ^24^ ( Figure 3). These observations suggest that there may still be explorable chemical space, which can be exploited to further increase Plk1-binding specificity. Notably, volasertib and its parental dihydropteridinone derivative, BI 2536 ^42^, do not interact with the Plk1-specific F58 and R134 residues but are in contact with the semi-specific R57, L132, and R136 residues and other neighboring residues (C67, L132, and F183) that are somewhat selective against non-Plks ( Figure 3). This finding explains in part why they exhibit a high level (~100–1,000-fold) of selectivity against other kinases but show less discriminatory activity against Plk1–3 (IC _50_ values of 0.83, 5, and 56 nM for Plk1, Plk2, and Plk3, respectively) ^25, 43^.

As noted above, one of the few differences between the ATP-binding pockets of Plk1 and Plk2 occurs near the hinge region (region II in Figure 3), where L132 of Plk1 corresponds to Y161 in Plk2. This difference has been exploited to generate inhibitors that demonstrate ~1,000-fold selectivity for Plk2 versus Plk1 ^44^. Such work highlights what can be achieved using structure-based drug design to take advantage of small differences in protein-binding sites. An additional effort to target the Plk1-specific F58 and R134 residues that lie at the mouth of the core ATP-binding pocket ( Figure 3) could lead to inhibitors with improved selectivity against other Plks. The generation of isoform-selective inhibitors is critically important to elucidate the biological roles of each individual Plk isoform, along with determining the importance of isoform selectivity in preventing the currently observed dose-limiting toxicities. Insights derived from such studies could further reveal the potential therapeutic value of targeting the catalytic domain of Plk1.

### Developing allosteric inhibitors

To ensure high selectivity, ATP-competitive small molecules must be designed to maximally engage in receptor-ligand interactions or sterically fill unoccupied space within defined binding pockets. In addition, because allosteric sites are generally less conserved than orthosteric sites, developing strategies to target allosteric sites, if available, can be potentially very useful in deriving specific inhibitors against a target. Close examination of the co-crystal structures of the Plk1 catalytic domain in complex with either ADP or volasertib reveals three ligand-binding regions (regions I, II, and III) that are important for achieving kinase selectivity ( Figure 3). Volasertib appears to utilize each of these orthosteric binding regions to achieve selectivity against most clinically important kinases. It is noteworthy that the region I located near the conserved Asp-Phe-Gly (DFG) motif contains a shallow space that could be potentially utilized to further improve the drug–target interaction. Interestingly, a dimethoxybenzeneethanamine derivative, SBE13, is suggested to bind to Plk1 in its “DFG-out” conformation and inhibit the enzyme’s catalytic activity with outstanding specificity ^45, 46^. An allosteric hydrophobic pocket generated in the catalytically inactive, DFG-out conformation of many Tyr kinases and, less frequently, Ser/Thr kinases has been successfully targeted to improve the selectivity of an inhibitor ^47– 49^. However, no structural information is yet available to verify whether Plk1 can indeed embrace a DFG-out conformation and generate an allosteric hydrophobic pocket that potentially allows an ATP-competitive inhibitor to trap the enzyme in a catalytically inactive state.

Another potentially appealing strategy toward achieving target-specific allosteric inhibition of Plk1 could be to block its essential substrate-binding interface. In principle, this may be approached by understanding the nature of the Plk1-substrate binding interactions that are critical for proper mitotic progression. ON01910, a substituted benzyl styryl sulfone, was initially reported as a non-ATP-competitive inhibitor of Plk1 that was thought to interfere with the substrate-binding site ^50^. However, subsequent studies showed that it possesses rather poor anti-Plk1 activity and specificity and inhibits multiple kinases ^43, 51– 53^. In a broad sense, the anti-PBD agents described below can be classified as target-specific allosteric inhibitors, which inhibit Plk1 function by binding to a specific site distant from the ATP-binding pocket.

## The PBD as an alternative target for anti-Plk1 drug discovery

The PBD is a functionally essential domain that binds to the enzyme’s physiological substrates and mediates the *cis-*acting phosphorylation of bound substrates by the *N*-terminal catalytic domain ^54^. A large body of evidence suggests that the Plk1 PBD contains a well-defined protein–protein interaction (PPI) pocket that can be inhibited by short peptides or peptide mimetics ^24^. Intriguingly, inhibition of Plk1 PBD function alone is sufficient for effectively imposing mitotic arrest and apoptotic cell death in cancer cells but not in normal cells ^20^. This suggests that targeting the PBD may serve as a promising alternative for developing anti-Plk1 therapeutics. It should be noted, however, that not all of Plk1’s substrates require a prior interaction with the PBD before being phosphorylated by its *N*-terminal catalytic domain ^54^. Therefore, inhibiting PBD function represents a target-restricted strategy that is designed to antagonize a subset of Plk1 functions dysregulated in cancer ^24, 54^.

## Advantages of targeting the Plk1 PBD

Unlike ATP-competitive inhibitors, whose specificities must be obtained against more than 500 other cellular kinases ^39^, PBD inhibitors target a structurally unique domain found in only four proteins (Plk1−3 and Plk5) ^24^. This greatly diminishes the likelihood of facing unwanted non-specific cross-reactivities. Another potential advantage of targeting the PBD is that while ATP-competitive inhibitors can abrogate all Plk1-dependent biochemical processes indiscriminately in both cancer and normal cells, PBD inhibitors interfere in only a subset of Plk1 functions that require a PBD-mediated biochemical step. Furthermore, since the Plk1 PBD can interact with a variety of structurally diverse phosphoepitope-containing proteins ^54^, it is reasonable to speculate that anti-PBD agents could potentially be optimized so that they selectively inhibit a subset of PBD-dependent interactions, which are enriched in biochemically rewired, Plk1-addicted cancer cells ^24^.

## Current status on the development of anti-Plk1 PBD agents

### Peptidomimetics

**Figure 4.**
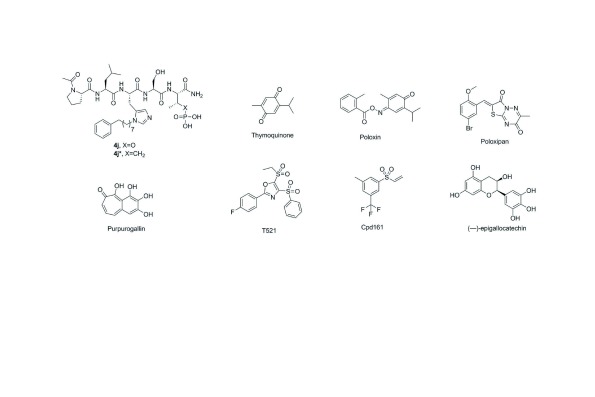
The structures of peptidomimetic and small molecule polo-box domain (PBD) inhibitors.

**Figure 5.**
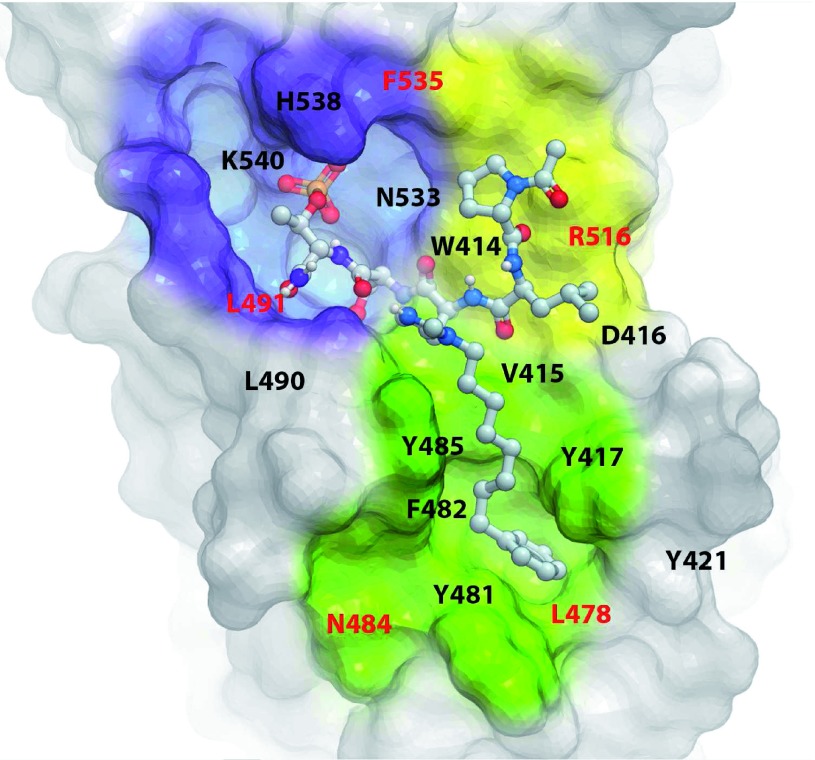
The structural model of polo-box domain (PBD) in complex with 4j. The X-ray cocrystal structure of the PBD + 4j complex (PDB: 3RQ7) shows a “Y-shaped” binding pocket composed of three discrete but interlinked binding modules—namely, a p-Thr/p-Ser-binding module (violet), a Pro-binding module (yellow), and a hydrophobic channel (green). Residues highlighted in red are specific to Plk1 PBD. Inhibitors designed to bind to more than one of the three binding modules could possess a superior binding specificity because of the specific requirement of the shape and geometrical arrangement of their binding moieties (see text for details).

Since the initial discovery of a pentameric p-Thr-containing peptide, PLHSpT, as a specific Plk1 PBD-binding ligand ^55^, several high-affinity peptide-derived inhibitors, including 4j ( Figure 4), have been generated ^56– 58^. These efforts have revealed that three distinct structural elements are critical for achieving high affinity and specific binding to the Plk1 PBD ( Figure 5). These are 1) a phosphoepitope-recognition pocket containing two basic residues (His538 and Lys540), which binds to Ser-Xxx motifs, where Xxx is p-Thr (or several-fold lower-affinity p-Ser) or a suitable anionic mimetic ^55, 59^, 2) an adjoining Pro-binding region that engages portions of the bound ligand *N*-proximal to the Ser-p-Thr dipeptide motif ^55^, and 3) a hydrophobic channel, which is capable of boosting the binding affinity ~500–1,000-fold without compromising Plk1 PBD specificity ^56– 59^. However, despite the fact that these peptide-based inhibitors exhibit extremely high affinity and specificity *in vitro*, their utility in cellular contexts is greatly decreased by poor membrane permeability and limited bioavailability. Efforts to increase cellular uptake have been directed at the reduction of anionic charge through charge masking and prodrug protection ^60^, macrocyclization ^61, 62^, and reducing peptide character ^63^.

### Small molecule inhibitors

Paralleling the development of peptidomimetics as described above are efforts to generate anti-Plk1 PBD agents that have yielded a wide range of small molecule inhibitors, including thymoquinone (TQ) and poloxin ^64^, poloxipan ^65^, purpurogallin (PPG) ^66^, T521 ^67^, Cpd161 ^68^, and (−)-epigallocatechin ^69^ ( Figure 4). As expected, if they interfered with the function of Plk1 PBD, these inhibitors induce mitotic block and apoptotic cell death, at least at the cultured cell level. While X-ray crystallographic data suggested the binding nature of how TQ and its analogue poloxime bound to the p-Thr/p-Ser-binding module ^70^, the assignment of these ligands within the phosphoepitope-recognition module appears to be disputable because of the poor resolution of the reported crystal structures. In addition, whether TQ and poloxin can be used as a template for structure-assisted drug design and hit-to-lead optimization remains in doubt because of their low Plk1 PBD specificity and non-specific alkylating activity with other cellular proteins ^68, 71, 72^. Indeed, covalent protein reactivity and other chemotypes, such as polyhydroxylated aromatic compounds (e.g. PPG), are thought to be some of the common artifacts that lead to the isolation of pan assay interference compounds (PAINS) ^73^ (as discussed in depth in a recent review ^74^).

## Future directions in anti-Plk1 PBD drug discovery

### Multidirectional approaches to identify a lead with druggable properties

Even though currently available peptide-derived and small molecule inhibitors have provided valuable information in understanding the nature of Plk1 PBD-dependent interactions and their cellular functions, it remains uncertain whether they can be used as leads for developing anti-PBD agents. Therefore, while these inhibitors are being further developed, additional endeavors could be considered in parallel in the hope of generating a new class of inhibitors with more druggable properties. Identifying promising leads possessing outstanding biochemical selectivity (i.e. specificity and affinity) and druggable physicochemical properties (e.g. solubility, bioavailability, chemical functionality, etc.) will likely be a critical step for successful lead optimization and ultimate discovery of anti-Plk1 PBD therapeutic agents.

### Exploiting the shape and geometrical arrangement of three adjacent binding modules of the Plk1 PBD-binding pocket to achieve high specificity

Now it is clear that, unlike the nondescript nature of many PPI surfaces, the Plk1 PBD furnishes a well-defined binding interface that may make it more amenable for structure-based drug discovery. Accumulated data show that the affinity and specificity of Plk1 PBD binding are dependent on the ability of a ligand to interact with three adjacently placed but biochemically distinct binding modules ^55, 56^ that collectively form a “Y-shaped” binding pocket (reviewed in Lee *et al*. ^24^) ( Figure 5). Future anti-Plk1 PBD drug discovery could take advantage of the Y-shaped interaction interface, so that ligands are optimized as three discrete but interconnected binding-module platforms.

Recent work has demonstrated that suitably designed p-Thr mimetic derivatives optimized for the phosphoepitope-recognition module can achieve several-fold enhancement in PBD-binding affinity ^75^. In addition, structural elaboration of hydrophobic channel-binding functionality and improvement of the interactions with Plk1-specific residues found in the surroundings of the hydrophobic channel have significantly increased overall ligand affinity and selectivity ^76, 77^. These findings suggest that the binding affinity and specificity of each of the three binding modules are determined independently of one another. Since a ligand capable of simultaneously binding to two or more binding modules must additionally conform to the exact geometrical arrangement of these modules, anti-Plk1 PBD agents generated through the Y-shaped, three-binding-module platform ( Figure 5) may likely possess uncommon physical shapes and chemical properties that may allow them to reach a high level of specificity. Multiple modular bindings discussed here may also help diminish the probability of developing drug resistance.

## Concluding remarks

Given the significant level of side effects associated with conventional MT-targeting anticancer therapeutics, developing an inhibitor against a mitosis-specific and cancer-cell-addicted target such as Plk1 may represent a promising strategy for the generation of a cancer-cell-specific therapeutic agent. One of the unique molecular features of Plk1 is that it offers, within one molecule, two independent drug targets—the *N*-terminal catalytic domain and the *C*-terminal PBD. The primary challenge facing currently available ATP-competitive inhibitors of Plk1 appears to be their dose-limiting toxicities. Although this hurdle can be conquered in principle by improving specificity or generating potentially more specific allosteric inhibitors as discussed above, considering extensive efforts made to date, this advancement may not be easily accomplished unless a breakthrough occurs. In this regard, it is quite intriguing to define the PBD as an alternative target for anti-Plk1 drug discovery. In addition to the superb binding specificity that PPI inhibitors can bring, Plk1 PBD inhibitors can provide an opportunity to selectively interfere with cancer-cell-enriched Plk1 PBD-binding targets. If developed, Plk1 PBD inhibitors with superior specificity can be used not only as a single therapeutic agent but also as an agent easily amenable for combination therapy with other anti-cancer therapeutics.

Significantly, the use of multiple inhibitors targeting different regions within one molecule is considered an effective way to eliminate drug resistance and to diminish the side effects of a high dose of a single agent. Therefore, using a Plk1 PBD inhibitor in combination with an ATP-competitive inhibitor, such as volasertib, could present a great treatment regimen that may achieve the maximum drug efficacy of both inhibitors while keeping volasertib at the highest acceptable dose that does not cause nonspecific cytotoxicity. Considering the success of anti-mitotic therapeutics and the remarkable advantages of targeting Plk1, there is ample reason to believe that further developing anti-Plk1 therapeutics may prove to be a worthwhile endeavor in the fight against cancer.
